# Immunosuppression and Chagas Disease: A Management Challenge

**DOI:** 10.1371/journal.pntd.0001965

**Published:** 2013-01-17

**Authors:** María-Jesús Pinazo, Gerard Espinosa, Cristina Cortes-Lletget, Elizabeth de Jesús Posada, Edelweiss Aldasoro, Inés Oliveira, Jose Muñoz, Montserrat Gállego, Joaquim Gascon

**Affiliations:** 1 Barcelona Centre for International Health Research (CRESIB), Hospital Clínic-Universitat de Barcelona, Barcelona, Spain; 2 Department of Autoimmune Diseases, Institut Clínic de Medicina i Dermatologia, Hospital Clinic i Provincial, Barcelona, Spain; 3 Department of Internal Medicine, Hospital General de L'Hospitalet, Barcelona, Spain; 4 Laboratori de Parasitologia, Facultat de Farmàcia, Universitat de Barcelona, Barcelona, Spain; Federal University of São Paulo, Brazil

## Abstract

Immunosuppression, which has become an increasingly relevant clinical condition in the last 50 years, modifies the natural history of *Trypanosoma cruzi* infection in most patients with Chagas disease. The main goal in this setting is to prevent the consequences of reactivation of *T. cruzi* infection by close monitoring. We analyze the relationship between Chagas disease and three immunosuppressant conditions, including a description of clinical cases seen at our center, a brief review of the literature, and recommendations for the management of these patients based on our experience and on the data in the literature. *T. cruzi* infection is considered an opportunistic parasitic infection indicative of AIDS, and clinical manifestations of reactivation are more severe than in acute Chagas disease. Parasitemia is the most important defining feature of reactivation. Treatment with benznidazole and/or nifurtimox is strongly recommended in such cases. It seems reasonable to administer trypanocidal treatment only to asymptomatic immunosuppressed patients with detectable parasitemia, and/or patients with clinically defined reactivation. Specific treatment for Chagas disease does not appear to be related to a higher incidence of neoplasms, and a direct role of *T. cruzi* in the etiology of neoplastic disease has not been confirmed. Systemic immunosuppressive diseases or immunosuppressants can modify the natural course of *T. cruzi* infection. Immunosuppressive doses of corticosteroids have not been associated with higher rates of reactivation of Chagas disease. Despite a lack of evidence-based data, treatment with benznidazole or nifurtimox should be initiated before immunosuppression where possible to reduce the risk of reactivation. Timely antiparasitic treatment with benznidazole and nifurtimox (or with posaconazole in cases of therapeutic failure) has proven to be highly effective in preventing Chagas disease reactivation, even if such treatment has not been formally incorporated into management protocols for immunosuppressed patients. International consensus guidelines based on expert opinion would greatly contribute to standardizing the management of immunosuppressed patients with Chagas disease.

## Introduction

Immunosuppression has gained increasing clinical relevance in the last 50 years. The list of neoplastic and systemic diseases that cause immunosuppression is increasing, as is the use of new immunosuppressive drugs. Furthermore, the exponential increase in the incidence of AIDS cases in the last 30 years has contributed to a significant increase in the number of immunosuppressed patients [1].

In most cases, immunosuppression modifies the natural history of other diseases, predisposing patients to the development or reactivation of opportunistic infections, with special, and often severe, clinical features [2]. The management of emerging infectious diseases, often unfamiliar to the clinician, becomes relevant in this context.

One of the most prevalent parasitic infections that has spread from Latin America to non-endemic areas such as the United States, Canada, and Europe is Chagas disease, or American trypanosomiasis [3], [4]. Its etiological agent, *Trypanosoma cruzi*, has not traditionally been considered an opportunistic agent, but in recent decades there have been many reports of reactivation in immunocompromised patients (mainly those with AIDS) [5]–[7].

With increasing migratory flows from poor, rural areas to large cities and developed, non-endemic countries, a growing number of patients with chronic Chagas disease are being diagnosed with other conditions that cause immunosuppression or require treatment with immunosuppressants; under these conditions, reactivation of the parasite is a likely outcome [5].

Reactivation is defined as an increase in parasitemia that can be detected by direct parasitological and/or PCR techniques, even in the absence of clinical symptoms [8]. The appearance of symptoms represents a worsening of the underlying disease. The most common symptoms of Chagas disease reactivation are subcutaneous nodules (chagoma), panniculitis, myocarditis with signs of heart failure, fever, meningitis, encephalitis, and stroke [9]–[12], but other symptoms, such as fatigue, anorexia, and diarrhea, are also seen [13]. In cases of reactivation, timely antiparasitic treatment with benznidazole has proven to be highly effective [14]–[16]. Several guidelines for the treatment of immunosuppressed patients with Chagas disease have been published recently [17]–[20], but there is no international consensus on the management of reactivation in these patients. While treatment with benznidazole is highly effective in reactivations [16], [21], it has not been formally incorporated into the management protocols of HIV/AIDS [22].

In this article we describe our experience with three immunosuppressive conditions (HIV infection, neoplastic disease, and systemic autoimmune disease) in patients with *T. cruzi* infection and provide some practical recommendations for the management of these patients based on our experience and a review of the literature. The aim is to contribute to the pool of knowledge in this area and to help improve the quality of care of patients with *T. cruzi* infection and any of the immunosuppressive conditions analyzed.

## Methods

We analyzed three immunosuppressive conditions in patients with *T. cruzi* infection: HIV/AIDS, neoplastic disease, and systemic autoimmune disease. For each category, we have included a review of the relevant literature, a description of all the clinical cases seen at our clinical care center between June 2007 and December 2010, and some recommendations for management based on our experience and the data in the literature.

The cases were diagnosed and treated at a specialist facility for the management of Chagas disease at the International Health Centre of Hospital Clínic in Barcelona, Spain. Screening for all patients includes *T. cruzi* and HIV tests, and general analyses.

Of 1,310 patients screened in the study period, *T. cruzi* infection was diagnosed in 715 patients. Of these, three had concomitant HIV infection, five had a neoplastic disease, and three had a systemic autoimmune disorder.

According to our in-hospital protocol for Chagas disease, all the patients signed informed consent before blood collection for diagnosis and before initiation of treatment with benznidazole. The therapeutic regimen in all cases was 5 mg/kg/day of benznidazole for 60 days.

The literature search was performed in the MEDLINE database using the terms *Chagas disease* and *T. cruzi*, with the subheadings *immunosuppression, HIV, systemic diseases, systemic lupus erythematosus*, *rheumatoid arthritis, reactivation, neoplasia, corticoids*, *chemotherapy*, *benznidazole, nifurtimox, PCR*, and *management*.

## Results and Discussion

### Coinfection with *T. cruzi* and HIV

#### Review of the literature

*T. cruzi* infection has been classified as an AIDS-defining opportunistic parasitosis by the US Centers for Diseases Control and Prevention since 2004 [23]. Considering the increasing number of cases of Chagas disease reactivation and the severity of the pathological manifestations associated with reactivation in patients with a low CD4^+^ T-cell count (usually <200 cells/mL) [9], [24]–[27], it is particularly important to consider *T. cruzi* infection as a potential opportunistic parasitic infection in patients in or from Chagas disease–endemic countries.

Even in asymptomatic patients, *T. cruzi* parasitemia is higher in those with concomitant HIV infection than in those without [28]–[30], and specific treatment with benznidazole or nifurtimox is recommended in such cases. The persistent immune activation induced by *T. cruzi* has been related to a sustained increase in HIV replication, with several studies showing an increase in plasma HIV-1 viral load in patients with parasitemia, indicating reactivation of *T. cruzi* infection [8], [15].

Reactivation in immunosuppressed patients is usually associated with more severe clinical manifestations (e.g., central nervous system [CNS] involvement, chagomas, meningoencephalitis, and severe myocarditis) than those observed in immunocompetent patients with acute Chagas disease [15], [26]. CNS infection by *T. cruzi* is more common than myocardial damage in HIV-immunosuppressed patients [6], [7], [15], [16]. Space-occupying lesions in the white matter of the brain are the most common clinical finding, but meningitis and meningoencephalitis may also occur [31]. CNS involvement was reported in 75%–80% of patients with concomitant *T. cruzi* and HIV infection between 1990 (when del Castillo et al. [19] described the first case of a young man with HIV infection and a lesion in the right frontal lobe with inflammatory perivascular infiltrates and clusters of *T. cruzi* amastigotes) and 2007 [13], [26], [32]–[40].

Cardiac compromise is the second most prevalent manifestation of reactivated Chagas disease, occurring in 25%–44% of patients coinfected with *T. cruzi* and HIV [26], [37], [41]–[43]. The most common cardiac manifestation is myocarditis, sometimes as a major presentation, but usually with CNS involvement masking heart involvement [29], [44]. Isolated reports of reactivation in other organs, such as the gastrointestinal tract, pericardium [26], peritoneum [45], skin [46], and cervix uteri [47], have been published.

#### Our experience

The epidemiological and clinical characteristics of the three patients with *T. cruzi* and HIV infection are shown in Tables 1 and 2, together with details of treatment and follow-up. Reactivation was not observed in any of the patients, but it should be noted that their CD4^+^ T-cell count remained above 200 cells/mL throughout follow-up.

**Table 1:** Patients with Chagas disease and HIV infection: demographic and clinical manifestations related to HIV infection

Patient	Epidemiological Features			Characteristics at Diagnosis				Characteristics at Follow-Up				
	Department, Country	Age	Sex	Year	Clinical Manifestations	CD4 T-Cell Count (Cells/µL)	HIV Viral Load (Copies/µL)	Opportunistic Infections	Latest CD4 T-Cell Count (Cells/µL)	Latest HIV Viral Load (Copies/µL)	Treatment	HIV Stage
1	Chuquisaca, Bolivia	32	F	2007	None	ND	ND	None	622	6.685	No	A1
2	Santa Cruz, Bolivia	46	M	2006	Herpes zoster	117	1,200,000	None	340	<60	ABV, 3TC, EFV	B2
3	Santa Cruz, Bolivia	28	F	2006	Genital herpes simplex	ND	ND	None	880	<60	EFV, EMC, TDF	B1

ND, no data; EFV, efavirenz; EMC, emtricitabine; TDF, tenofovir; ABV, abacavir; 3TC, lamivudine.

**Table 2:** Patients with Chagas disease and HIV infection: demographic and clinical manifestations related to Chagas disease

Patient	Epidemiological Features			Chagas Disease							
	Department, Country	Age	Sex	Risk Factors for *T. cruzi* Infection	Year of Diagnosis	Clinical Stage	Treatment (m/year)	Follow-Up (months)	Outcome of Specific Treatment (during Follow-Up)	PCR Results	
										Before Treatment	During Follow-Up
1	Chuquisaca, Bolivia	32	F	VC, VT, TF	2004	CCC (KI^a^)	BZD (02/2009)	36	No changes in clinical stage	Positive (08/05/2008)	NA
2	Santa Cruz, Bolivia	46	M	VC	2008	Indeterminate stage^b^	BZD (02/2009)	40	No changes in clinical stage	Positive (11/10/2008)	Negative (11/02/2009)
3	Santa Cruz, Bolivia	28	F	VC	2008	Indeterminate stage	BZD (10/2008)	38	No changes in clinical stage	ND	ND

a Mild chronic cardiac disease according to Kuschnir classification [67].

b Relevant comorbidities: asymptomatic strongyloidosis diagnosed in 2006 treated with albendazole and ivermectin; type 2 diabetes mellitus and high blood pressure, well controlled by oral antidiabetic and antihypertensive drugs; macrocytic anemia due to folic acid deficiency.

VC, contact with vector; VT, mother with *T. cruzi* infection; TF, transfusion in endemic area; BZD, benznidazole; CCC, chronic cardiac disease stage; ND, no data; NA, not accomplished.

#### Recommendations for management

HIV-positive patients with moderate immunosuppression and latent Chagas disease are usually asymptomatic, and parasitemia normally increases before the appearance of symptoms [45]. Nevertheless, treatment with benznidazole or nifurtimox should be initiated as soon as any symptoms of *T. cruzi* infection reactivation are noted, even in the absence of an increase in parasitemia [7], [15], [31], [48]. Direct microscopic examination of blood and cerebrospinal fluid may reveal the presence of *T. cruzi* trypomastigotes, which usually, but not always, precedes clinical manifestations [49]. Parasitological and/or PCR follow-up are thus mandatory in patients with HIV infection [50].

In some patients with myocardial involvement, the clinical manifestations are heart failure and arrhythmias, but findings from autopsy studies suggest that a significant proportion of patients with Chagas disease and HIV/AIDS have clinically silent cardiac disease, even though they have inflammatory foci associated with *T. cruzi*–infected cardiomyocytes [21], [40].

Brain imaging techniques are theoretically useful for exploring CNS involvement, but considering the lack of specificity of brain imaging and the large variability in the space occupied by *T. cruzi*–induced lesions in individuals with AIDS, a brain biopsy provides better guarantees of accurate diagnosis and appropriate therapy. Histopathologic examination of the space-occupying lesions of the brain shows severe inflammation in association with heavily parasitized glial cells and occasionally neurons [21].

Specific treatment with benznidazole or nifurtimox is recommended when there is strong evidence of *T. cruzi* infection reactivation. The recommendation grade is AII (treatment should always be offered) in patients with reactivated *T. cruzi* infection and HIV/AIDS or another immunosuppressive disorder, and BII (treatment should generally be offered) in those with impending immunosuppression [51]. Despite a lack of evidence-based data, *T. cruzi*–specific treatment should be considered at the moment of HIV diagnosis, prior to severe immunosuppression, in order to prevent or minimize the risk of Chagas disease reactivation.

The duration of Chagas therapy has not been standardized in HIV/AIDS, but some groups recommend maintaining treatment throughout immunosuppression [13], as is done in many patients with Chagas disease who undergo organ transplantation. Specific chemotherapy in Chagas disease is still unsatisfactory, and while benznidazole and nifurtimox can be effective in reducing parasitemia, controlling clinical manifestations, and reducing mortality (and thereby improving prognosis) during reactivations in immunocompromised individuals, they have frequent adverse side effects and limited capacity for achieving a parasitological cure; such facts should be taken in account to decide the length of treatment [29], [52].

#### Summary

*T. cruzi* infection is an opportunistic parasitic infection indicative of AIDS. Clinical manifestations are more severe in reactivation than in acute Chagas disease, and usually involve the CNS. Parasitemia should be considered the most important defining feature of reactivation, because it usually increases before the onset of symptoms. Treatment with benznidazole and/or nifurtimox is strongly recommended in cases of clinical and/or parasitological suspicion of reactivation.

### Pharmacological Immunosuppression: Neoplastic Disease in Individuals with *T. cruzi* Infection

#### Review of the literature

Little is known about the relationship between the incidence of neoplastic disease and *T. cruzi* infection, or about whether the immune response induced by this infection modifies the development or location of neoplasms.

The relationship between immunosuppression and neoplastic disease in patients with *T. cruzi* infection has been analyzed from three perspectives: 1) neoplastic disease as a potential immunosuppressive condition that increases the likelihood of reactivation of infection; 2) neoplastic disease occurring as a result of treatment with benznidazole, either alone or combined with immunosuppressive drugs; and 3) neoplastic disease as a condition that is potentially more common in patients with Chagas disease.

In our opinion, the most relevant issue regarding the association between neoplastic disease and Chagas disease is the potential for *T. cruzi* reactivation and the management of this reactivation in order to prevent serious consequences.

Based on clinical observations, some authors have speculated as to whether benznidazole treatment in immunosuppressed patients might actually cause neoplasms [10], [53]. However, several clinical studies with long follow-up have found no evidence of a higher incidence of histological signs of malignancy in such patients [11], [52], [54]. The conclusions of these studies are clear: benznidazole is not related to the development of neoplasms and the association between benznidazole and other immunosuppressive drugs does not contribute to an increased incidence of neoplastic disease.

A higher prevalence of esophageal carcinoma and uterine cervix leiomyoma has been reported in patients with chronic Chagas disease than in individuals without *T. cruzi* infection [55]. Esophageal carcinoma is more common in patients with Chagas disease and megaesophagus than in those without Chagas disease [56], but a similar prevalence has been reported in patients without megaesophagus, regardless of whether or not they have Chagas disease [56]. The association between esophageal carcinoma and Chagas disease in patients with megaesophagus is probably a consequence of the chronic esophagitis caused by alimentary stasis. Although some clinical studies have claimed an association between megacolon due to Chagas disease and colon cancer [57], [58], an experimental study in mice with chagasic megacolon, where fecal stasis was observed, failed to reveal a higher incidence of colon cancer [59]

#### Our experience

In our series, four patients with *T. cruzi* infection had bone marrow cancer and one had breast adenocarcinoma. The characteristics of the patients and the diseases are summarized in Table 3. Two of the patients died, one due to myeloid acute leukemia and the other due to lymph node metastasis from primary breast adenocarcinoma. None of the patients had clinical evidence of reactivation during follow-up.

**Table 3:** Demographic and clinical manifestations of patients with *T. cruzi* infection and neoplastic disease.

	Epidemiological Features			Neoplastic Disease			Chagas Disease							Outcomes
							Characteristics at Diagnosis				Follow-Up			
Patient	Department, Country	Age	Sex	Year of Diagnosis	Type	Therapy	Risk Factor	Year	Treatment (m/year)	Clinical Form	Clinical Manifestations	Time (months)	PCR Follow-Up	
1	Chuquisaca, Bolivia	52	F	2008	Myeloid acute leukemia M1	IDICE-G	VC	1997	ND	KI	No	36	Negative (01/16/2006; 10/14/2008)	Alive/NR
2	Chuquisaca, Bolivia	50	F	2006	B cell high-grade non Hodgkin lymphoma	R-MEGACHOP/R-ESHAP	VC	2006	BZD (02/2007)	IND	No	48	Negative (01/24/2007; 06/26/2007)	Alive/NR
3	Arequipa, Perú	50	F	2005	Myeloid acute leukemia secondary to gastric adenocarcinoma	• IDICE-G• MTX• Mitox +ARA-C (intensification)	VC	2005	BZD (ND/2006)	IND	No	40	Negative (01/19/2006; 03/26/2007)	Death
4	Cochabamba, Bolivia	44	M	2010	Multiple myeloma (IgG kappa)	• VBCMP• Zoledronate	VC	2005	BZD (04/2010)	IND	No	12	Positive (03/17/2010;04/21/2010); negative (05/04/2010)	Alive/NR
5	Cochabamba, Bolivia	37	F	2007	Breast adenocarcinoma (pT4N3, RH, HER2)	Docetaxel + radical mastectomy	VC, VT	2007	Not indicated	CCC (K III)^a^	Syncope	24	NA	LN progression, death

a Severe chronic cardiac disease according to Kuschnir classification.

ND, no data; NA, not accomplished; NR, no reactivations; VC, contact with the vector; VT, mother with *T. cruzi* infection; IND, indeterminate stage of Chagas disease; CCC, chronic cardiac disease stage; R-MEGACHOP, Rituximab-MEGACHOP; R-ESHAP, Rituximab-ESHAP; IDICE-G, idarubicin, ARA-C, etoposide; MTX, intrathecal methotrexate; Mitox, mitoxantrone; VBCMP or M2, vincristine, carmustine, melphalan, cyclophosphamide, prednisone; LN: lymph node.

#### Recommendations for management

Reactivation of *T. cruzi* infection must be considered in patients with chronic Chagas disease and neoplastic disease requiring intensive or long-term pharmacological immunosuppression. It is not possible, however, to make a general evidence-based recommendation for the management of such patients or for the prevention of reactivation. The risks of toxicity associated with trypanocidal treatment are well known and efficacy in patients with chronic disease is variable and limited, which renders risk/benefit analyses difficult. Based on reports in the literature and on our experience, it would seem reasonable to administer trypanocidal treatment only to asymptomatic immunosuppressed patients with detectable parasitemia (Strout method or PCR) at the moment of evaluation, or to patients with clinically defined reactivation. Trypanocidal treatment at the moment of diagnosis and prior to immunosuppression might minimize the risk of reactivation, but this indication is controversial and is not supported by evidence-based data. Experience in this area is limited to isolated case reports and series.

#### Summary

Specific treatment for Chagas disease does not appear to be related to a higher incidence of neoplasms, and a direct role of *T. cruzi* in the etiology of neoplastic disease has not been confirmed. Reactivation, however, might be an unavoidable consequence of immunosuppressive treatment. Patients with *T. cruzi* infection who need immunosuppressive therapy must thus be closely followed, and treatment with benznidazole or nifurtimox is strongly recommended in cases of confirmed reactivation.

### Systemic Autoimmune Disease and Chagas Disease

#### Review of the literature

Systemic autoimmune diseases such as systemic lupus erythematosus (SLE) and rheumatoid arthritis (RA) are considered rare in patients with *T. cruzi* infection, but this may be due to the underdiagnosis of autoimmune diseases in low-income areas/countries. The coexistence of Chagas disease and these two conditions has been rarely reported in the scientific literature, with only 12 cases of SLE [60]–[62] and one of RA described to date [63].

Barousse et al. [60] reported on a series of ten patients with SLE and *T. cruzi* infection treated with nifurtimox. Five of the patients were receiving concomitant immunosuppressive therapy and three of these died. Reactivation was not identified in these patients (autopsy did not show *T. cruzi* amastigotes, so Chagas disease was not identified as cause of death for any of them) or in the remaining seven patients monitored during follow-up. Dos Santos-Neto et al. [61] described a patient with mesangial proliferative lupus glomerulonephritis and Chagas disease treated with prednisone (1 mg/kg) and monthly pulses of cyclophosphamide (1 g/m^2^ of body surface). They reported reactivation in the form of CNS involvement and high parasitemia determined by xenodiagnosis, and treated successfully with benznidazole. After 8 years of follow up, the patient died of generalized lupus vasculitis. The twelfth case of SLE in association with Chagas disease was described by our group in 2010 [62]. In the only case of concomitant Chagas disease and RA in the literature [63], the patient developed polymyositis 28 years after the diagnosis of RA. Benznidazole was not prescribed. The detection of parasites in the muscle lesions suggested Chagas disease etiology, and the worsening clinical condition and disease progression could have been related to the use of corticosteroids.

Two studies have provided valuable information about the use of corticosteroids at immunosuppressive doses in patients with *T. cruzi* [64], [65]. Rassi et al. [65] described the effectiveness of benznidazole in reducing reactivation rates in patients with *T. cruzi* infection treated with corticosteroids, but they also described higher rates of parasitemia that appeared to be related to higher doses of corticosteroids. Nevertheless, in all the cases, there were other causes of immunosuppression besides the long-term use of corticosteroids. There has just been one case reported of reactivation of Chagas disease in which corticosteroids were the only immunosuppressive factor: that of a child receiving dexamethasone for a cranial traumatism [66]. Currently, there is no firm evidence of an association between immunosuppressive doses of corticosteroids alone and higher rates of *T. cruzi* reactivation [64].

#### Our experience

Our experience with Chagas disease in association with autoimmune diseases consists of three patients with SLE and one with RA (Tables 4 and 5). All the patients received benznidazole from the moment of diagnosis, and no episodes of reactivation were observed during follow-up. In one patient with SLE and chronic Chagas disease (the one coming from Misiones, Argentina) who was diagnosed with lupus nephritis requiring immunosuppressive treatment, response to benznidazole was not achieved, but subsequent treatment with posaconazole led to a successful resolution of the infection. In fact, despite maintenance of immunosuppressive therapy due to renal involvement, parasitemia became negative and no episodes of reactivation were observed in a follow-up period of 36 months.

**Table 4:** Patients with systemic autoimmune diseases: demographic and clinical manifestations related to autoimmune disease

Patient	Autoimmune Disease	Epidemiological Features			Characteristics at Diagnosis			Characteristics at Follow-Up	
		Department, Country	Age	Sex	Year	Clinical Manifestations	Treatment	Remission	Maintenance Treatment
1	SLE	Santa Cruz, Bolivia	40	F	2007	• Nephritis class IV• AIHA, arthritis• Oral ulcers	Pulses of MP followed by PDN 1 mg/kg/day plus 6 monthly pulses of CYC followed by 2 quarterly pulses of CYC (750 mg/m^2^)	Complete	• PDN 2.5 mg/day• AM 360 mg/12 h• HDX 200 mg/day
2	SLE	Cochabamba, Bolivia	46	F	2008	Skin involvement	PDN 10 mg/day	Complete	PDN 10 mg/day
3	SLE	Misiones, Argentina	44	F	2007	• Nephritis class IV• Arthritis, malar rash	Pulses of MP followed by PDN 1 mg/kg/day plus 6 monthly pulses of CYC (750 mg/m^2^)	Complete	• PDN 2.5 mg/day• AZA 100 mg/day• HDX 200 mg/day
4	RA	Cochabamba, Bolivia	36	F	2010	Polyarthritis	None	Arthralgia	None

AM, acid mycophenolic; AZA, azathioprine; CYC, cyclophosphamide; HDX, hydroxychloroquine; MP, methylprednisolone; PDN, prednisone; RA, rheumatoid arthritis; SLE, systemic lupus erythematosus.

**Table 5:** Patients with systemic autoimmune diseases: demographic and clinical manifestations related to Chagas disease

Patient	Autoimmune Disease	Epidemiological Features			Chagas Disease					
		Department, Country	Age	Sex	Risk Factors for *T. cruzi* Infection	Year of Diagnosis	Clinical Features	Treatment (year)	Follow-Up (months)	PCR during the Follow-Up
1	SLE	Santa Cruz, Bolivia	40	F	VC	2000	CCC (K I)^a^	BZD	36	Negative (05/28/2007; 07/22/2008)
2	SLE	Cochabamba, Bolivia	46	F	VC	2005	Indeterminate stage	BZD	48	ND
3	SLE	Misiones, Argentina	44	F	VC, TF	1987	Indeterminate stage	BZD, Posaconazole	36	Positive (05/17/2007); negative^b^ (07/02/2010)
4	RA	Cochabamba, Bolivia	36	F	VC	1997	Chagas chronic digestive	BZD	36	Negative (06/30/2008)

a Mild chronic cardiac disease according to Kuschnir classification [67].

b PCR was negative in nine determinations between 03/18/2008 and 07/02/2010, after treatment with posaconazole.

VC, contact with the vector; TF, transfusion in endemic area; BZD, Benznidazole; CCC, chronic cardiac disease stage; ND, no data.

#### Recommendations for management

The most relevant consequences of continuous immunosuppressive treatment in patients with systemic autoimmune disease and Chagas disease is the risk of reactivation of *T. cruzi* infection and the development of severe, chronic forms of the disease. Unfortunately, guidelines for the management of *T. cruzi* infection in this setting do not exist. In immunosuppressed patients, trypanocidal treatment with benznidazole or nifurtimox is indicated (BII recommendation) [51]. This treatment should be initiated before immunosuppression where possible to decrease the risk of reactivation. The use of benznidazole in patients who require long-term corticosteroid therapy is controversial due to the lack of supporting evidence.

#### Summary

Clinicians should bear in mind that the natural course of *T. cruzi* infection can be modified by other diseases or their treatment. Immunosuppressive doses of corticosteroids have not been associated with higher rates of reactivation of Chagas disease. Furthermore, there have been no reports of a relationship between Chagas disease reactivation and other immunosuppressive drugs used in the treatment of systemic autoimmune diseases, and it has not been shown that these diseases have a direct role in the progression of *T. cruzi* infection.

## Final Considerations

The number of patients under immunosuppressive therapy is increasing and timely management is important to reduce the risk of comorbidities. The main goal in *T. cruzi–*infected patients with an immunosuppressive condition is to prevent serious consequences of reactivation of *T. cruzi* infection by close monitoring, and in the event of reactivation, to start early treatment with benznidazole and/or nifurtimox to minimize clinical complications. Currently there is no international consensus on how to manage the clinical situations described in this article, and it is difficult to develop evidence-based recommendations due to the small number of Chagas disease patients diagnosed with an immunosuppressive condition besides HIV. As in other clinical situations, consensus guidelines based on expert opinion could greatly contribute to standardizing the management of patients with *T. cruzi* infection and a concomitant immunosuppressive condition.

**Learning Points**

- Early diagnosis of *T. cruzi* infection in immunosuppressed individuals is extremely important, and should be assessed prior to immunosuppressive treatment.
- In *T. cruzi*–infected patients with an immunosuppressive condition, the main goal is to prevent reactivation by close monitoring.
- Parasitemia measured by PCR techniques is recommended in immunosuppressed patients during follow-up.
- In cases of reactivation, early treatment with benznidazole and/or nifurtimox is strongly recommended, and posaconazole should be indicated when this fails.

**Key Papers in the Field**

- de Freitas VL, da Silva SC, Sartori AM, Bezerra RC, Westphalen EV, et al. (2011) Real-time PCR in HIV/*Trypanosoma cruzi* coinfection with and without Chagas disease reactivation: association with HIV viral load and CD4 level. PLoS Negl Trop Dis 5(8): e1277. doi:10.1371/journal.pntd.0001277
- Cordova E, Boschi A, Ambrosioni J, Cudos C, Corti M (2008) Reactivation of Chagas disease with central nervous system involvement in HIV-infected patients in Argentina, 1992–2007. Int J Infect Dis 12: 587–592.
- Galhardo MC, Martins IA, Hasslocher-Moreno A, Xavier SS, Coelho JM, et al. (1999) Reactivation of *Trypanosoma cruzi* infection in patients with acquired immunodeficiency syndrome. Rev Soc Bras Med Trop 32: 291–294.
- Nishioka S de A (2000). Benznidazole in the primary chemoprophylaxis of the reactivation of Chagas' disease in chronic chagasic patients using corticosteroids at immunosuppressive doses: is there sufficient evidence for recommending its use? Rev Soc Bras Med Trop 33: 83–85.
- Bern C, Montgomery SP, Herwaldt BL, Rassi A Jr., Marin-Neto JA, et al. (2007) Evaluation and treatment of Chagas disease in the United States: a systematic review. JAMA 298: 2171–2181.
